# Epigenetically regulated miR-145 suppresses colon cancer invasion and metastasis by targeting LASP1

**DOI:** 10.18632/oncotarget.11919

**Published:** 2016-09-09

**Authors:** Wei Wang, Gang Ji, Xin Xiao, Xu Chen, Wei-Wei Qin, Fan Yang, Yu-Fang Li, Lin-Ni Fan, Wen-Jin Xi, Yi Huo, Wei-Hong Wen, An-Gang Yang, Tao Wang

**Affiliations:** ^1^ State Key Laboratory of Cancer Biology, Department of Immunology, Fourth Military Medical University, Xi'an, Shaanxi, PR China; ^2^ Department of Digestive Diseases, Xijing Hospital, Fourth Military Medical University, Xi'an, Shaanxi, PR China; ^3^ Department of Orthopedics, Xijing Hospital, Fourth Military Medical University, Xi'an, Shaanxi, PR China; ^4^ Department of Pathology, Fourth Military Medical University, Xi'an, Shaanxi, PR China; ^5^ Department of Medical Genetics and Developmental Biology, Fourth Military Medical University, Xi'an, Shaanxi, PR China

**Keywords:** colorectal cancer, metastasis, miR-145, LASP1, histone methylation

## Abstract

MiR-145 is a tumor-suppressive microRNA that participates in the malignant progression of colorectal cancer (CRC). Although miR-145 has been reported to inhibit proliferation and to induce apoptosis of CRC cells, the reports about its role in invasion and metastasis are controversial. The regulation of miR-145 its own expression also requires further elucidation. In this study, we firstly found that miR-145 is markedly downregulated in the metastatic tumors of CRC patients. Then through gain- and loss-of function studies, we demonstrated that miR-145 suppresses the invasion and metastasis of CRC cells. We also provided experimental evidences which include direct binding assays and verifications on tissue specimens to confirm that LIM and SH3 protein 1 (LASP1) is a direct target of miR-145. Furthermore, we identified the core promoter regions of miR-145 and observed the cooperation between histone methylation and transcription factors through binding to these core promoter regions to regulate the expression of miR-145 in CRC cells. Our study provides an insight into the regulatory network in CRC cells, thus offering new targets for treating CRC patients.

## INTRODUCTION

The role of microRNAs (miRNAs) in human malignancies has been intensively studied in recent years [1-3]. MiR-145, as an anti-tumor microRNA, has been shown to be under-expressed and to repress various target genes to inhibit the malignant processes of several types of tumors [4-7]. In colorectal cancer (CRC), miR-145 has been shown to suppress a number of oncogenes, such as c-Myc [8], DNA fragmentation factor 45 [9], and P70 ribosomal S6 kinase [10], to participate in the growth, cell cycle distribution, apoptosis, and angiogenesis of CRC cells. However, there are different views on the role of miR-145 in invasion and metastasis of CRC cells. A few studies have reported miR-145 as an oncogenic regulator during these processes [11-13], but more researches found miR-145 as a tumor suppressor inhibiting invasion and metastasis of CRC cells [14-19]. In addition to its role and mechanisms in CRC invasion and metastasis, the regulation of miR-145 expression has not been fully elucidated. Dysregulation of miRNA expression in different cancers can be ascribed to improper binding of transcription factors on response elements of the promoter regions and epigenetic changes including aberrant DNA methylation and histone modification [20]. Notably, the cooperation between transcription factors and histone methylation could represent a common regulation of miRNA expression [21, 22]. However, reviewing previous studies reporting the upstream regulation of miR-145 [7, 8, 18, 23-25], we found that neither histone methylation nor its cooperation with transcription factors has been studied.

In this study, we confirmed the suppressive role of miR-145 on CRC invasion and metastasis and investigated the mechanisms both *in vitro* and *in vivo*. Importantly, we are the first to demonstrate that miR-145 directly suppresses LASP1 to inhibit the invasion and metastasis of CRC and also the first to find that a histone methylation involved mechanism, together with the binding of transcription factors on the promoter region of miR-145, may co-regulate the expression of miR-145 in CRC cells.

## RESULTS

### MiR-145 is downregulated in metastatic CRC tumors and inhibits the invasion and metastasis of CRC cells *in vitro*

**Figure 1:**
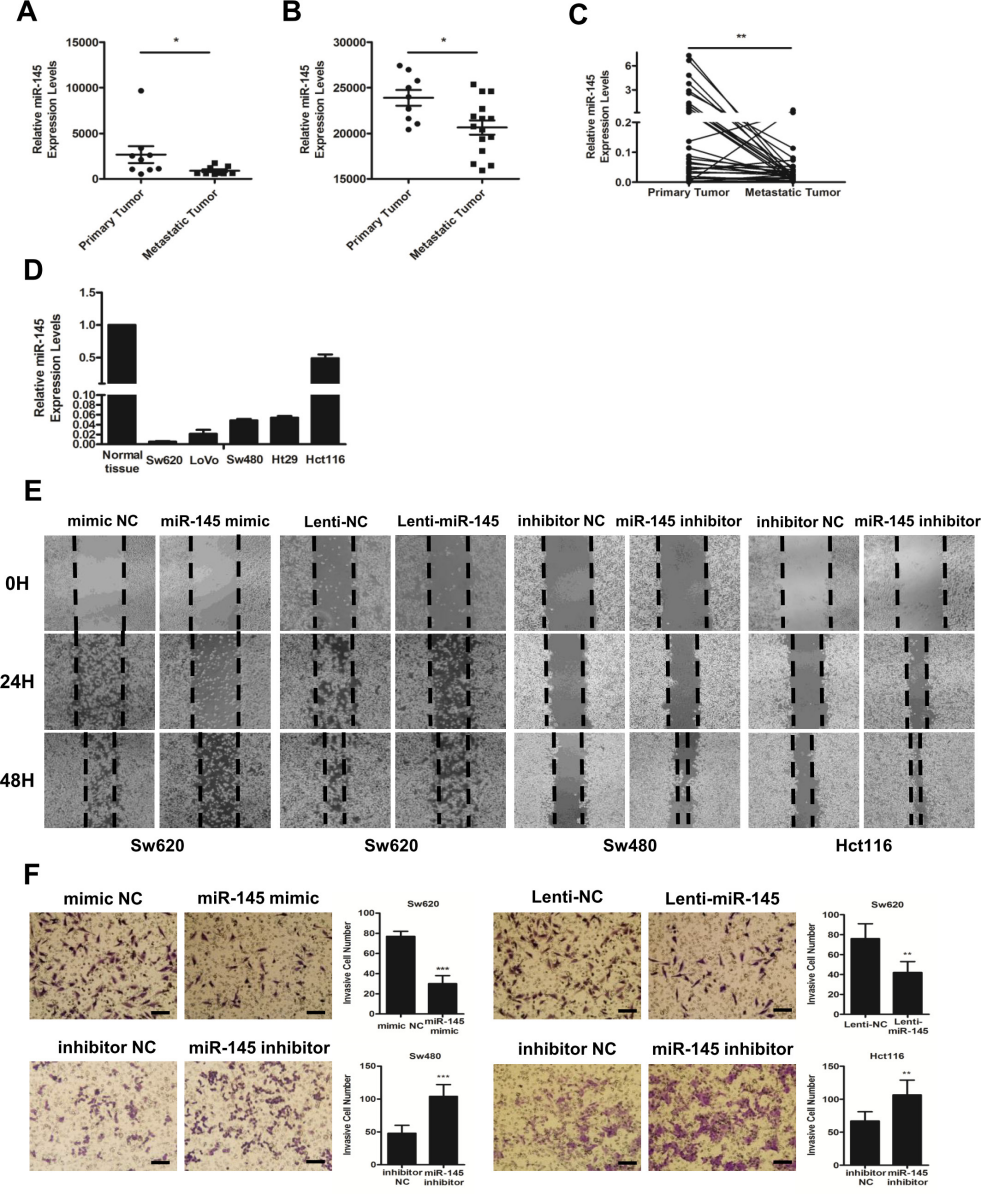
MiR-145 is downregulated in metastatic CRC tumors and inhibits the invasion and metastasis of CRC cells *in vitro* **A, B.** miR-145 expression levels from the GSE44121 (A) and GSE54088 (B) GEO Datasets. Data are represented as the mean ± s.e.m. * *P* < 0.05 (Student's t-test) **C.** miR-145 is differentially expressed in paired primary and metastatic tumor tissues from 33 CRC patients analyzed by qRT-PCR. ** *P* < 0.01 (Paired t-test). **D.** qRT-PCR analyses of miR-145 in five CRC cell lines (Hct116, Ht29, LoVo, Sw480 and Sw620) compared with the mean rate of miR-145 expression in 33 normal tissues. Data represent the mean ±s.e.m. of three independent experiments. **E, F.** mimic NC/miR-145-mimic-treated Sw620 cells, Lenti-NC/Lenti-miR145-treated Sw620 cells and inhibitor NC/miR-145 inhibitor-treated Sw480 and Hct116 cells were subjected to wound-healing assays (E) and Matrigel invasion assays (F). Scale bars are 50 μm. Data are represented as the mean ± s.d. of three independent experiments. ** *P* < 0.01 and *** *P* < 0.001 (Student's t-test).

Two studies from the Gene Expression Omnibus (GEO) Datasets (GSE44121, Figure 1A and GSE54088, Figure 1B) showed that compared with primary tumors, the expression of miR-145 was significantly decreased in the corresponding metastatic tumors of CRC patients. To further verify this finding, we collected the paraffin-embedded tissue specimens of matched adjacent normal mucosa, primary tumors and metastatic lymph nodes or hepatic tumors from 33 CRC patients from XiJing Hospital (Supplementary Table 1). QRT-PCR analyses showed that compared with that in primary tumor tissues, the expression of miR-145 was pronouncedly downregulated in metastatic tumor tissues (Figure 1C). Accordingly, while miR-145 was downregulated in all CRC cell lines, its expression level was decreased to a greater extent in the more aggressive Sw620 cell line (Figure 1D), indicating a tumor-suppressive, and particularly anti-metastatic role for miR-145 in CRC cells.

To verify the anti-metastatic role of miR-145, we conducted gain- and loss- of function experiments in Sw620, Sw480, and Hct116 cells. The transient transfection of miR-145 mimics and the stable transfection of miR-145 lentiviral particles (Lenti-miR-145) markedly impaired the ability of Sw620 cells to migrate into a monolayer of wounded cells (Figure 1E) and to invade the membrane of trans-well chambers (Figure 1F) compared with mimic NC/Lenti-NC-transfected Sw620 cells. The transfection of miR-145 inhibitors markedly increased the migration and invasion of Sw480 and Hct116 cells during wound-healing (Figure 1E) and Matrigel invasion assays (Figure 1F) compared with the inhibitor NC-transfected cells. These findings confirmed that miR-145 inhibits the invasion and metastasis of CRC cells *in vitro*, as suggested by data from CRC patients and cell lines.

### MiR-145 inhibits EMT and the invasion and metastasis of CRC cells *in vivo*

**Figure 2:**
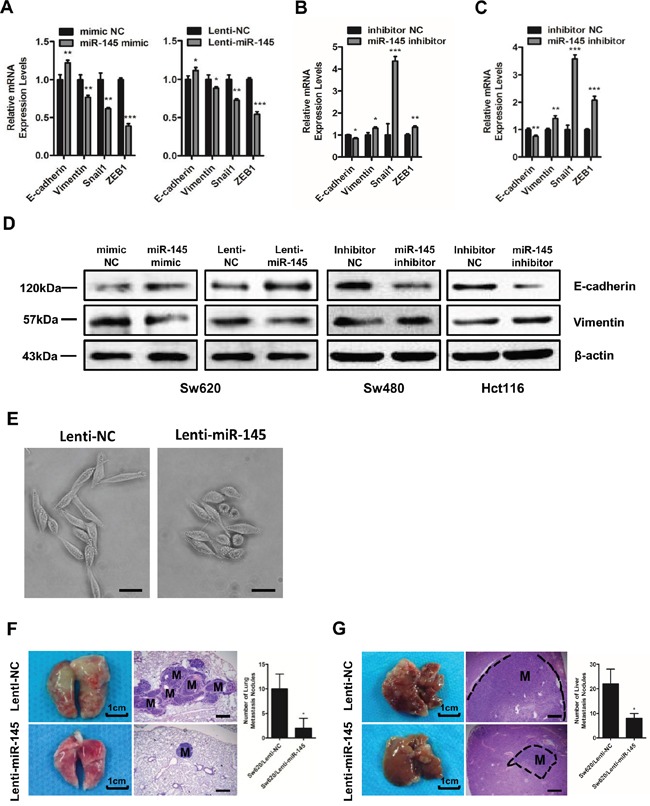
MiR-145 inhibits EMT and the invasion and metastasis of CRC cells *in vivo* **A-C.** qRT-PCR analyses of EMT markers in mimic NC/miR-145 mimic-treated Sw620 cells, Lenti-NC/Lenti-miR145-treated Sw620 cells (A) and inhibitor NC/miR-145 inhibitor-treated Sw480 (B) and Hct116 cells (C). Data represent the mean ± s.e.m. of three independent experiments. **D.** Western blot analyses of E-cadherin and Vimentin in mimic NC/miR-145 mimic-treated Sw620 cells, Lenti-NC/Lenti-miR145-treated Sw620 cells and inhibitor NC/miR-145 inhibitor-treated Sw480 and Hct116 cells. **E.** Observation of Sw620/Lenti-NC cells and Sw620/Lenti-miR-145 cells under light microscopy. Scale bars are 50 μm. **F, G.** Lungs (F) and livers (G) of nude mice in the Sw620/Lenti-NC and Sw620/Lenti-miR-145 groups 6 weeks after tail vein injection (F) and 5 weeks after spleen injection (G) as labeled by H&E staining of the metastatic tumor tissues. The numbers of metastatic lung and liver nodules per nude mouse were counted under a microscope. M: metastatic lesion. Scale bars are 500 μm. Data represent the mean ± s.d. of six replicates in each group. * *P* < 0.05, ** *P* < 0.01 and *** *P* < 0.001 (Student's t-test).

To further prove the anti-metastatic role of miR-145 in CRC cells, we tested the alterations of epithelial-mesenchymal transition (EMT) markers following intracellular increase or decrease of miR-145 level. Results showed that miR-145 inhibitor treatment resulted in significant changes towards EMT at the mRNA levels of EMT markers such as epithelial protein E-cadherin and mesenchymal markers of Vimentin, Snail1 and zinc finger e-box binding homeobox (ZEB1) and at the protein levels of both E-cadherin and Vimentin in Sw480 (Figure 2B&2D) and Hct116 (Figure 2C&2D) cells compared with control cells. We observed the opposite expression patterns of these markers in miR-145 mimic- and Lenti-miR-145-treated Sw620 cells (Figure 2A&2D). Moreover, we observed that compared with Sw620/Lenti-NC cells which grew as loosely packed spindle-like fibroblastic cells, the Sw620/Lenti-miR-145 cells appeared to grow more tightly in contact with each other, morphologically demonstrating an anti-EMT role of miR-145 (Figure 2E). To test the anti-metastatic role of miR-145 *in vivo*, we injected Sw620/Lenti-NC and Sw620/Lenti-miR-145 cells into the tail vein and spleen of nude mice respectively. Consistent with our visual observations, H&E analyses and nodule counts under the microscope confirmed the presence of larger and more metastatic nodules in the lungs (Figure 2F) and livers (Figure 2G) of mice from the Sw620/Lenti-NC groups, whereas smaller and less metastatic nodules were observed in mice from the Sw620/Lenti-miR-145 groups (Figure 2F&2G). These data show that miR-145 inhibits the invasion and metastasis of CRC cells both *in vitro* and *in vivo*.

### LASP1 is a direct target of miR-145

**Figure 3:**
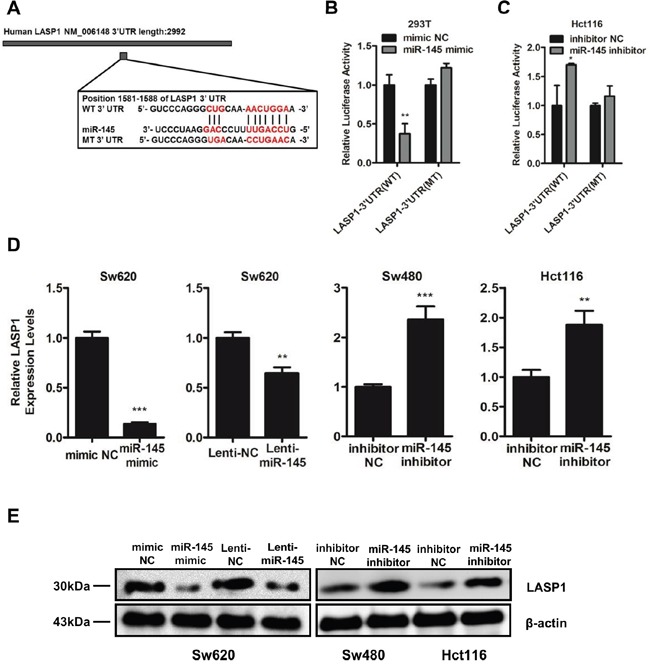
LASP1 is a direct target of miR-145 **A.** Diagram of LASP1 3′UTR containing the putative conserved target sequence for miR-145. WT: wild type; MT: mutant type. **B, C.** Results of luciferase reporter assays in 293T (B) and Hct116 cells (C) co-transfected with LASP1 WT/MT 3′UTR vectors and miR-145 mimic (B) or miR-145 inhibitor (C) as indicated for 48 h. Data are represented as the mean ± s.d. of three independent experiments. **D.** qRT-PCR analyses for LASP1 mRNA levels following transfection of miR-145 mimic and Lenti-miR-145 into Sw620 cells and miR-145 inhibitor into Sw480 and Hct116 cells. Data are represented as the mean ± s.e.m. of three independent experiments. **E.** Western blot analyses for LASP1 protein levels following transfection of miR-145 mimic and Lenti-miR-145 into Sw620 cells and miR-145 inhibitor into Sw480 and Hct116 cells. *P < 0.05, ** *P* < 0.01 and *** *P* < 0.001 (Student's t-test).

Based on bioinformatic predictions via the publicly available algorithms [26], we focused on the invasion- and metastasis-related genes among the putative target genes including Fascin-1 (FSCN1), LIM and SH3 protein 1 (LASP1), ADAM Metallopeptidase Domain 17 (ADAM17), Neural Precursor Cell Expressed Developmentally Down-Regulated 9 (NEDD9), mucin 1 (MUC1) and so on. Previous study has reported FSCN1 as one of the targets of miR-145 [16]. As LASP1 forms complexes with FSCN1 to serve as indispensable actin filament-bundling proteins that stabilize the lamellipodia of cancer cells [27] and has already been demonstrated to promote the metastasis of colorectal cancer [28, 29], we predicted that LASP1 is another potential target of miR-145. To verify this prediction, we investigated the ability of miR-145 to directly target the 3′-untranslated region (3′UTR) of LASP1 by cloning the wild-type (WT 3′UTR) and mutant (MT 3′UTR) miR-145 target sequences of the 3′UTR region of LASP1 into a pGL3-mcs2 vector (Figure 3A). Luciferase reporter assays indicated a significant decrease in the luciferase activity of miR-145 mimic- and WT 3′UTR vector-co-transfected 293T cells (Figure 3B) and a marked increase in miR-145 inhibitor- and WT 3′UTR vector-co-treated Hct116 cells (Figure 3C) compared with the NC group, whereas the luciferase activity did not change in the MT 3′UTR vector-treated groups (Figure 3B&3C). Furthermore, LASP1 expression decreased in Sw620 cells when treated with miR-145 mimics or Lenti-miR-145 particles (Figure 3C&3D), whereas LASP1 expression increased at both the mRNA (Figure 3C) and protein (Figure 3D) levels in miR-145 inhibitor-treated Sw480 and Hct116 cells. These results indicate that miR-145 directly suppresses LASP1 expression by binding to its 3′UTR.

### LASP1 is involved in miR-145-mediated tumor-suppressive effects

**Figure 4:**
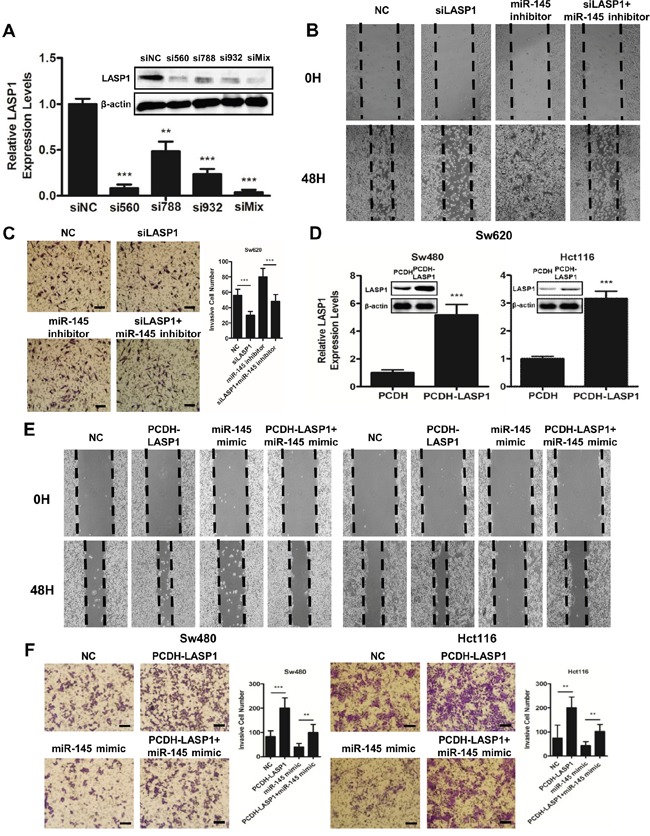
LASP1 is involved in miR-145-mediated tumor-suppressive effects **A.** Efficiencies of transfection of siLASP1 package (si560, si788 and si932 alone and a mixture together, siMix) into Sw620 cells was detected by qRT-PCR and Western blot analyses. **B, C.** miR-145 inhibitor- and siLASP1 (siMix)-treated Sw620 cells were subjected to wound-healing assays (B) and Matrigel invasion assays (C). **D.** Efficiencies of transfection of PCDH-LASP1 into Sw480 and Hct116 cells were detected by qRT-PCR and Western blot analyses. **E, F.** miR-145 mimic- and PCDH-LASP1-treated Sw480 and Hct116 cells were subjected to wound-healing assays (E) and Matrigel invasion assays (F). Scale bars are 50 μm. Data represent the mean ± s.e.m. (A&D) or mean ± s.d. (C&F) of three independent experiments. ** *P* < 0.01 and *** *P* < 0.001 (Student's t-test).

To determine the function of LASP1 in CRC cells, we co-transfected Sw620 cells with miR-145 inhibitor and a mixture of three LASP1 small interfering RNAs (siLASP1, shown as siMix in Figure 4A). We also simultaneously co-transfected Sw480 and Hct116 cells with miR-145 mimic and PCDH-LASP1 vector which contained the coding sequence of LASP1 (Figure 4D). The silencing of LASP1 in Sw620 cells suppressed cell invasion and metastasis (Figure 4B&4C), while the overexpression of LASP1 in Sw480 and Hct116 cells significantly enhanced the metastatic and invasive capacities of CRC cells (Figure 4E&4F). Furthermore, the silencing of endogenous LASP1 partially abolished the miR-145 inhibitor-induced invasiveness (Figure 4B&4C), whereas the exogenous expression of LASP1 partially neutralized the miR-145 mimic-mediated inhibition of cell migration and invasion in wound-healing and Matrigel invasion assays (Figure 4E&4F). These results further suggested that miR-145 inhibits the invasion and metastasis of CRC cells, at least in part by downregulating LASP1 expression.

### Expression level of LASP1 inversely correlates with miR-145 level *in vitro*, *in vivo* and in CRC patients' tissue specimens

**Figure 5:**
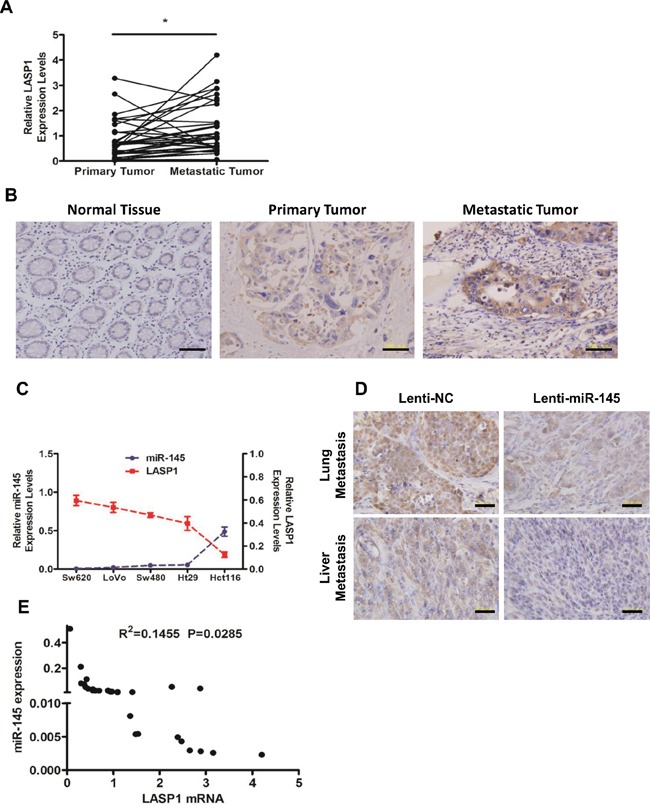
Expression level of LASP1 inversely correlates with miR-145 level *in vitro*, *in vivo* and in CRC patients' tissue specimens **A, B.** Expression levels of LASP1 in matched (normal mucosa) primary tumor and metastatic tumor tissues from 33 CRC patients as detected by qRT-PCR analyses (A) and IHC staining (B, from No. 5 of the 33 patients). * *P* < 0.05 (Paired t-test). Scale bars are 100 μm. **C.** Expression levels of miR-145 and LASP1 in Hct116, Ht29, LoVo, Sw480 and Sw620 cells were detected by qRT-PCR. Data represent the mean ± s.e.m. of three independent experiments. **D.** Representative photographs of anti-LASP1 IHC staining of lung (upper) or liver (lower) metastatic tumor tissues from Sw620/Lenti-NC and Sw620/Lenti-miR-145 nude mice groups. Scale bars are 50 μm. **E.** The correlation of LASP1 mRNA and miR-145 in metastatic tumor tissues from 33 CRC patients. The Pearson product-moment correlation coefficient and significance level are indicated.

We performed qRT-PCR analyses (Figure 5A) and IHC staining (Figure 5B) on tissue specimens from the 33 CRC patients. Results showed that the expression of LASP1 was significantly upregulated in metastatic tumor tissues compared with primary tumor tissues in both mRNA and protein levels (Figure 5A&5B). In tests to further confirm the correlation between levels of miR-145 and LASP1, we found that in CRC cell lines, the LASP1 mRNA levels inversely correlated with miR-145 levels (Figure 5C). IHC staining of metastatic tumor tissues from nude mice revealed that the protein levels of LASP1 in metastatic tumor cells from the Sw620/Lenti-miR-145 group were decreased significantly compared with those from the Sw620/Lenti-NC group (Figure 5D). In addition, the LASP1 mRNA levels were found inversely correlated with miR-145 levels in the metastatic tumor specimens from the 33 CRC patients (Figure 5E). Taken together, these results from experiments *in vitro*, *in vivo* and in CRC patients' tissue specimens indicate that the expression level of LASP1 inversely correlates with miR-145 level, adding strong evidences to the direct suppression of miR-145 on LASP1 expression.

### A histone methylation involved mechanism regulates the expression of miR-145 through its core promoter regions

**Figure 6:**
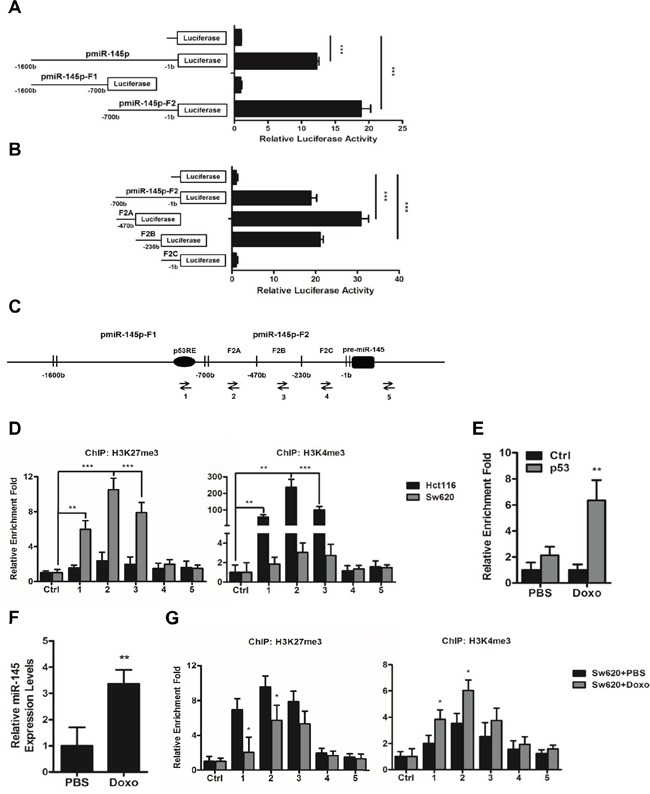
A histone methylation involved mechanism regulates the expression of miR-145 through its core promoter regions **A, B.** The putative promoter region of miR-145 (pmiR-145p), its truncated fragments pmiR-145p-F1 and pmiR-145p-F2 (A), and the three sub-fragments of pmiR-145p-F2, F2A, F2B, and F2C (B) were cloned into pGL3-Basic vector, and their promoter activities were detected by luciferase reporter assays. **C.** Arrows indicate positions of Primers 1-5 used for ChIP assays of miR-145 promoter region, of which Primer 2, 3 and 4 specifically amplifies F2A, F2B, and F2C and Primer 1 amplifies the previously reported p53 response element (pP53RE). **D, G.** ChIP assays of the miR-145 core promoter regions using rabbit IgG (Ctrl), H3K27me3-specific and H3K4me3-specific antibodies in untreated Hct116 and Sw620 cells (D) and Sw620 cells after 16 h treatment of PBS or Doxo (1ug/ml) (G). **E.** ChIP assays of the P53RE region in miR-145 promoter using mouse IgG1 (Ctrl) and p53-specific antibodies in Sw620 cells after 16 h treatment of PBS or Doxo (1ug/ml). **F.** Expression levels of miR-145 in Sw620 cells after 16 h treatment of PBS or Doxo (1ug/ml). Data are represented as the mean ± s.d. of three independent experiments. *P < 0.05, ** *P* < 0.01 and *** *P* < 0.001 (Student's t-test).

To study the activity of the putative promoter region of miR-145 [8], we cloned the upper 1.6kb sequence from pre-miR-145 (pmiR-145p), and its truncated fragments, 900-bp long pmiR-145p-F1 and 700-bp long pmiR-145p-F2 into pGL3-Basic vector respectively. Data from luciferase reporter assays carried in Hct116 cells revealed a notable promoter activity of the whole pmiR-145p while the F2 fragment showed an obvious promoter activity (Figure 6A). After sub-truncating pmiR-145p-F2 into three sub-fragments of equal lengths, F2A, F2B, and F2C respectively, we found the F2A and F2B sub-fragments held the most obvious promoter activity (Figure 6B). To further study the regulatory mechanism involved in these core promoter regions, we applied chromatin immunoprecipitation (ChIP) assays with primers to specifically amplify the F2A, F2B, and F2C sub-fragments and a region which was previously reported to harbor a p53 response element (p53RE) [8] (Figure 6C). Results showed that in Sw620 cells expressing the lowest level of miR-145, significant enrichment of trimethylation of histone H3 at lysine 27 (H3K27me3) were found in the F2A and F2B core promoter regions (Figure 6D), whereas in Hct116 cells expressing the highest level of miR-145, the core promoter regions were occupied by trimethylation of histone H3 at lysine 4 (H3K4me3) (Figure 6D), suggesting the expression of miR-145 was possibly activated or inhibited by methylation of histones on core promoter regions in the upper regulatory sequence of pre-miR-145.

In addition, the similar enrichment patterns of H3K27me3 and H3K4me3 in the p53RE region in both of the cell lines (Figure 6D) attracts our attention. We treated Sw620 cells with 1.0ug/ml doxorubicin (Doxo) for 16 h to induce endogenous expression of p53 [8] and observed significant increase of p53 enrichment in the p53RE region (Figure 6E), as well as increased miR-145 level in Sw620 cells (Figure 6F), which was in accordance with previous report that p53 directly pinds to the p53RE in miR-145 promotor to induce miR-145 [8]. Furthermore, we detected significantly decreased enrichment of H3K27me3 and increased enrichment of H3K4me3 in the p53RE region following induction of p53 expression by Doxo treatment (Figure 6G). Similar alterations of H3K27me3 and H3K4me3 enrichment were also detected in the F2A and F2B core promoter regions (Figure 6G). Taken together, these results suggested that histone methylation may cooperate with binding of transcription factors on the core promoter regions in determining the level of miR-145 in CRC cells.

## DISCUSSION

Our present study reveals that miR-145 inhibits the invasion and metastasis of CRC cells both *in vitro* and *in vivo* (Figures 1&2), which was in accordance with most of the previous studies [14-19]. Analyzing the studies which gave the opposite conclusion [11-13], we deduce that using small sample size of tumor tissues [12], having enough sample size but not paired primary and metastatic tumor tissues from the same CRC patients [13], and statistically analyzing primary tumor tissues with or without lymph node metastases but not matched primary and real metastatic tumor tissues from CRC patients [11] might be the reasons why these reports found an oncogenic role of miR-145 in the metastasis and invasion of CRC. In our study, we used strictly matched adjacent normal mucosa, primary tumors and metastatic lymph nodes or hepatic tumors from 33 CRC patients for the miR-145 tests to minimize these biases. We further demonstrated that LASP1 is a direct target gene of miR-145. LASP1 was initially identified from a cDNA library of metastatic axillary lymph nodes of patients with breast cancer [30, 31], and is reportedly overexpressed in several types of metastatic cancers including CRC [29, 32]. Its role in TGFβ-Mediated EMT of CRC cells has also been proved and this pro-EMT effect is realized by regulating S100A4 expression [28]. Recent study also proved the vital role of LASP1 in tumor metastasis by working together with Vimentin in hepatocellular carcinoma (HCC) cells [33]. In our study, we demonstrated that as a direct target of miR-145 (Figures 3&5), LASP1 negatively participates in miR-145-mediated metastasis-suppressive effects (Figure 4).

Previous studies have shown that miR-145 could be epigenetically silenced by DNA methylation of its promotor region, leading to aggressive malignancies [7, 23] such as brain metastasis of lung cancer [24]. Also, several transcription factors have been found to negatively [25] or positively [8, 18] regulate the expression of miR-145 by directly binding to response elements in the promoter regions. These studies give us a clue of the upstream regulation of miR-145 expression in carcinogenesis, but also reveal the gaps in finding the role of another important part of epigenetic regulation, the histone methylation in determining miR-145 level. As the primary markers studied in this mechanism regulating miRNAs, enrichment of H3K27me3 in the promotor region represents the contraction of chromatin and the following inactivation of miRNA transcription [34], whereas that of H3K4me3 represents the dispersal of chromatin and the following activation of miRNA transcription [35, 36]. Here in our study, we firstly confirmed the core promoter regions of miR-145 and then observed the role of histone methylation in regulating miR-145 expression (Figure 6A-6D). We also observed the orchestration between H3K4me3 and binding of p53 on the core promoter regions in increasing the miR-145 level in CRC cells (Figure 6E-6G). These findings add more evidence to the co-regulation pattern consisting of both histone methylation and transcription factors in the regulation of miRNAs in various cancer cells [21, 22].

In conclusion, by finding a novel target, LASP1, in the suppression of miR-145 on CRC invasion and metastasis and observing a novel mechanism, the cooperation between histone methylation and transcription factors, in the regulation of miR-145 during CRC carcinogenesis, we provided an insight into the regulatory network in CRC cells and offered new targets for treating CRC patients.

## MATERIALS AND METHODS

### Human CRC specimens

Paraffin-embedded human tissue specimens of matched adjacent normal mucosa, primary tumors and metastatic lymph nodes or hepatic tumors from 33 CRC patients (Supplementary Table 1) were collected from Xijing Hospital of Digestive Disease affiliated to the Forth Military Medical University (FMMU). Patients included must have a pathological diagnose of CRC in both the primary and metastatic tumor sites and must have undergone elective surgery of both the primary and metastatic tumors in Xijing Hospital of Digestive Disease during 2010-2015. The study was approved by the Medical Ethics Committee of FMMU, and written informed consents were obtained from all patients. Careful microdissection was performed.

### Cell culture and co-transfection

Hct116, Sw480, Sw620 and 293T cell lines were obtained from the Cell Bank of Chinese Academy Sciences (SIBS, Shanghai, China) and used for the *in vitro* experiments. All the cell lines used were authenticated and tested in the cell bank after the bank bought them from American Type Culture Collection (ATCC, Manassas, VA, USA) before sale. Hct116 cells were cultured in McCoy's 5A (Modified) Medium (Gibco, Los Angeles, CA, USA); Sw480 and Sw620 cells were cultured in Leibovitz′s L-15 Medium (Gibco), and 293T cells were cultivated in Dulbecco's Modified Eagle Medium (DMEM, Gibco). All of the media were supplemented with 10% fetal bovine serum (FBS) (Gibco). All cells were incubated at 37°C in a 5% CO_2_ atmosphere. CRC cells were transfected with plasmids and oligonucleotides using Lipofectamine^®^ 2000 reagent (Invitrogen, Carlsbad, NM, USA) at final concentrations of 2 μg/ml and 50 nM respectively for 48 h, according to the manufacturer's instructions. The transfection efficiency was monitored by qRT-PCR.

### RNA isolation, reverse transcription and quantitative real-time polymerase chain reaction (qRT-PCR)

Total RNA, including miRNA, was extracted and purified using an miRNeasy FFPE Kit (Qiagen, Hilden, Germany) for paraffin-embedded tissue specimens and TRIzol reagent (Invitrogen, Carlsbad, NM, USA) for CRC cells. Reverse transcription reactions for mRNA and miRNA were then performed with PrimeScript™ RT Master Mix and SYBR^®^ PrimeScript™ miRNA RT-PCR Kit (TaKaRa Bio Group, Shiga, Japan), respectively. qRT-PCR analyses were performed using SYBR^®^ Premix Ex Taq™ II (TaKaRa) on a Bio-Rad CFX96 system (Bio-Rad, CA, USA) according to the manufacturer's protocols. β-actin and U6 RNA were used as internal loading controls for mRNA and miRNA analyses, respectively. All samples were normalized to internal controls, and fold changes were calculated via relative quantification (2-ΔΔCT). The primers used for qRT-PCR analyses are in Supplementary Table 2.

### Oligonucleotides, plasmids construction and stable transfection

All synthetic miRNA mimics and miRNA inhibitors, including negative control (NC), miR-145 mimic, inhibitor NC and miR-145 inhibitor, were purchased from GenePharma (Shanghai, China). Silencer Select Negative Control (siNC), and the package of LASP1 siRNA (siLASP1, a mixture of si560, si788 and si932 as shown in Supplementary Table 3) was also obtained from GenePharma. The coding sequence of LASP1 was amplified from Sw620 cell cDNA using the following paired primers: 5′-GCTCTAGAATGCTTCCATTGCGAG-3′ and 5′-CGAATTCTCAGATGGCCTCCACGTA-3′. The resulting 0.6 kb fragment was cloned into the XbaI and EcoRI sites of PCDH, yielding the PCDH-LASP1 overexpression vector. Lentiviral particles for hsa-miR-145 miRNA were bought from GeneCopoeia (Guangzhou, China). Sw620 cells were transfected with lentiviral particles of NC/miR-145 (Lenti-NC/Lenti-miR-145, Titer: 1.02×10^9^ copies/ml) according to the manufacturer's instructions. The sequences of the oligonucleotides are in Supplementary Table 3.

### Wound-healing assay and matrigel invasion assay

For the wound-healing assays, wound closures were observed by taking photographs under a microscope 0, 24 and 48 hours (h) after scratching. Matrigel invasion assays were performed with Matrigel (BD Biosciences, Heidelberg, Germany) and 8-μm, 24-well trans-well chambers (Millipore, Billerica, MA, USA) following the manufacturer's instructions. Cells (1×10^5^) in 200 μl of serum-free medium were added to the upper chamber and cultured for 48 h. Migrated cells were stained with 0.1% crystal violet for 10 minutes at room temperature, and photographs were taken of ten randomly selected fields of fixed cells. The cells were then counted in high-power fields by light microscopy.

### Animals, *in vivo* tumor metastatic assays

Six-week-old, male Nude nu/nu mice were maintained in a sterile facility in racks supplied with high-efficiency particulate-filtered air. The animals were fed an autoclaved laboratory rodent diet. The mice in this study were purchased from the Experimental Animal Centre of the Forth Military Medical University (FMMU), which is certified by the Shaanxi Provincial Bureau of Science. All animal experiments complied with ethical regulations and humane treatment and were approved by the Medical Ethics Committee of FMMU. An estimated sample size of at least six mice per group was chosen to ensure adequate power to detect a pre-specified effect size. During the tumor metastasis test, mice which died earlier than the endpoint time were excluded from the analysis.

To evaluate the lung and liver metastatic potential of cancer cells *in vivo*, 5 × 10^6^ Sw620/Lenti-NC or Sw620/Lenti-miR-145 cells in 200 μl of serum-free medium were injected into nude mice through the tail vein (n = 6 per group) and the same amount of cells in 50 μl of serum-free medium were injected into the spleens (n = 6 per group), respectively. Whole-body optical images were visualized to monitor primary tumor growth and the formation of metastatic lesions (Lighttools). Five weeks later, the spleen injected mice were sacrificed, and six weeks later, the tail vein injected mice were sacrificed. Individual organs from the mice were removed, and metastatic tissues were analyzed with H&E staining.

### Bioinformatics and accession numbers

Potential miRNA targets were predicted and analyzed using the publicly available algorithms: TargetScan (http://www.targetscan.org/), PicTar (http://pictar.mdc-berlin.de/) and miRanda (http://www.microrna.org/microrna/home.do). The Gene Expression Omnibus (GEO) accession numbers for the miRNA expression data in Figure 1A&1B are GSE44121 and GSE54088.

### Luciferase reporter assay

Human LASP1-3′ untranslated region (3′UTR) reporter plasmids containing the putative binding sequence of miR-145 (wild-type, WT) and its identical sequence with a mutation in the miR-145 seed sequence (mutant, MT) were amplified by PCR, inserted between the EcoRI and EcoRV restriction sites of the pGL3-msc2 reporter vector (Promega, Madison, WI, USA), and validated by sequencing. 293T and Hct116 cells were plated at 3 × 10^4^ cells/well in a 48-well plate and transfected with LASP1-3′UTR WT/MT vectors (100 ng/well) and mimic NC/miR-145 mimic or inhibitor NC/miR-145 inhibitor (50 nM). To study the activity of the putative promoter region of miR-145, we cloned the upper 1.6kb sequence from pre-miR-145, its truncations Fragment 1 (pmiR-145p-F1), Fragment 2 (pmiR-145p-F2) and the sub-truncations of pmiR-145p-F2 (F2A, F2B, F2C) into pGL3-Basic vector respectively. A pTK-luc (Renilla) luciferase vector (5 ng/well) was transfected into 293T or Hct116 cells for normalization. The luciferase activity was measured 48 h after transfection using the Dual-Luciferase Reporter Assay System (Promega) according to the manufacturer's instructions. To analyze the data, the raw luciferase activity was first normalized by the internal transfection control Renilla and then divided by the average value of the NC group. The primers used for constructions of reporter plasmids are in Supplementary Table 4.

### Western blot analysis

The protein concentrations of the cell lysates were quantified with the BCA method. The proteins were separated on an SDS/PAGE gel and then transferred onto a nitrocellulose (NC) membrane. The NC membrane was incubated with primary antibody overnight at 4°C followed by an incubation with secondary antibody for 1 h at room temperature. The experiment was repeated at least three times. Antibodies against LASP1 (1:5000 dilution, Proteintech 10515-1-AP), E-cadherin (1:100 dilution, Santa Cruz Biotechnology sc-7870), Vimentin (1:100 dilution, Santa Cruz Biotechnology sc-66002) were used, and β-actin (1:2000 dilution, Sigma-Aldrich A1978) was used as a loading control. Goat anti-rabbit and goat anti-mouse immunoglobulin HRP-linked F(ab)2 fragments (1:5000 dilution, Akea, Guangzhou, China) were used as secondary antibodies. The signals were detected by an enhanced chemiluminescence system (ECL System, Alpha Innotech, CA, USA) according to the manufacturer's instructions.

### Immunohistochemistry

For tissue immunohistochemistry (IHC) staining, FFPE sections of normal or tumor tissues from 33 CRC patients and nude mice were deparaffinized with xylene (3 x 15 min), followed by treatment with serial dilutions of ethanol. The antigens were unmasked by boiling the slides (95 - 99°C) for 15 min in 10 mM sodium citrate at pH 6.0. The sections were cooled to room temperature, immersed in 3% H_2_O_2_ for 10 min, and blocked for 1 h with blocking solution (5% normal goat serum in PBS). Primary antibodies against LASP1 (1:200 dilution, Proteintech 10515-1-AP, Chicago, IL, USA) was diluted in Antibody Diluent for IHC (Beyotime, Shanghai, China) and incubated with the sections overnight at 4°C. The sections were then incubated with biotinylated secondary antibody for 10 min (Maxim, Fuzhou, China) and with streptavidin horseradish peroxidase (HRP, Maxim) for another 10 min at room temperature. Subsequently, the sections were stained with a DAB Substrate Kit (Maxim) for 1-2 min and counterstained with hematoxylin (Maxim). Finally, the tissue sections were dehydrated and mounted in Eukitt medium. Images were captured with a light microscope and processed with identical settings.

### Chromatin immunoprecipitation

Chromatin immunoprecipitation (ChIP) assays were performed using the Chromatin Immunoprecipitation Assay Kit (Millipore) according to the manufacturer's instructions. Briefly, proteins were cross-linked to DNA by formaldehyde. The cells were then lysed and sonicated to shear the DNA into 200 to 500 bp fragments. Following an overnight incubation with the immunoprecipitating antibodies [against normal rabbit immunoglobin G (IgG, 2 μg, #2729), Cell Signaling Technology, Denver, MA, USA, against H3K27me3 (5 μg, #9733), Cell Signaling Technology, against H3K4me3 (5 μg, ab8580), Abcam, Cambridge, UK, against normal mouse immunoglobin G1 (IgG1, 2 μg, #5415), Cell Signaling Technology, and against p53 (5 μg, sc-126), Santa Cruz Biotechnology, Dallas, TX, USA] and 1 h of incubation with Protein A/G PLUS-Agarose Imminoprecipitation Reagent (sc-2003, Santa Cruz Biotechnology) at 4°C, the immunoprecipitates were subjected to multiple washes. The DNA recovered after reversion of the protein-DNA cross-links with NaCl was incubated with proteinase K and subsequently extracted with phenol-chloroform and precipitated with ethanol. qRT-PCR analyses using different sets of primers to amplify the miR-145 regulatory region were performed using the immunoprecipitated DNA (20 ng/reaction), and the relative fold-enrichment for each set of primers was normalized to that of the IgG (Ctrl) group. The primer sequences for miR-145 regulatory region analyzed are in Supplementary Table 5.

### Statistical analysis

Statistical analysis was performed using SPSS 15.0 software (SPSS Inc., USA). Data are expressed as the mean ± standard deviation (s.d.) or mean ± standard error of the mean (s.e.m.) from at least three separate experiments. Two-tailed Student's t-tests were used to evaluate statistical significance between two independent groups of samples. The significance of correlations between mRNA and miRNA level was judged via a test statistic based on Pearson product-moment correlation coefficient. Differences were considered significant when *P < 0.05, **P < 0.01 and ***P < 0.001.

## SUPPLEMENTARY MATERIALS TABLES


