# Supplementary material for: Epigenetically regulated miR-145 suppresses colon cancer invasion and metastasis by targeting LASP1
Source: Oncotarget. 2016 Sep 9;7(42):68674–87. doi: 10.18632/oncotarget.11919 (PMC5356582; doi:10.18632/oncotarget.11919)
Supplement: Supplementary file 1 [file oncotarget-07-68674-s001.pdf]

Epigenetically regulated miR-145 suppresses colon cancer invasion and metastasis by targeting LASP1

SUPPLEMENTARY TABLES

Supplementary Table S1: Information of CRC patients collected for clinical studies

	Total n=33 (%)
**Age (years)**	
**≤ 60**	21 (63.6)
**> 60**	12 (36.4)
**Gender**	
**Female**	15 (45.5)
**Male**	18 (54.5)
**Tumor Status**	
**T3**	18 (54.5)
**T4**	15 (45.5)
**Tumor Metastasis**	
**Lymphatic**	29 (87.9)
**Hepatic**	4 (12.1)

Clinical information of the 33 patients whose tissue specimens were collected to investigate the expression levels of miR-145.

Supplementary Table S2: Primers used for qRT-PCR analyses in this study

Gene	Forward (5'-3')	Reverse (5'-3')
Hsa-miR-145	GTCCAGTTTTCCCAGGAATCCC	RR716, SYBR® PrimeScript™ miRNA RT-PCR Kit, TaKaRa
U6	CTCGCTTCGGCAGCACA	AACGCTTCACGAATTTGCGT
E-cadherin	TGCCCAGAAAATGAAAAAGG	GTGTATGTGGCAATGCGTTC
Vimentin	GAGAACTTTGCCGTTGAAGC	GCTTCCTGTAGGTGGCAATC
Snail1	ACAAGCACCAAGAGTCCG	ATGGCAGTGAGAAGGATGTG
ZEB1	GACAGTGTTACCAGGGAGGAGCA	TTCAGGTGCCTCAGGAAAAATGA
LASP1	CCTTCTTCTGCCACTTCTGC	CAACAGGTCTCCCATTCCAG
β-actin	TGGCATCCACGAAACTACC	GTGTTGGCGTACAGGTCTT

Supplementary Table S3: Sequences of the oligonucleotides in this study

Oligonucleotides	Sense (5'-3')	Antisense (5'-3')
mimic NC/siNC	UUCUCCGAACGUGUCACGUTT	ACGUGACACGUUCGGAGAATT
miR-145 mimic	GUCCAGUUUUCCCAGGAAUCCCU	GGAUUCCUGGGAAAACUGGACUU
inhibitor NC	CAGUACUUUUGUGUAGUACAA	
miR-145 inhibiotr	AGGGAUUCCUGGGAAAACUGGAC	
siLASP1(si560)	CCGAGCUCCAGAGAAUCAATT	UUGAUUCUCUGGAGCUCGGTT
siLASP1(si788)	AGUCCUAUGGUGGCUACAATT	UUGUAGCCACCAUAGGACUTT
siLASP1(si932)	UCGUCAACGUGCAGCAGAUTT	AUCUGCUGCACGUUGACGATT

Supplementary Table S4: Primers used for construction of plasmids in this study

Gene	Plasmid	Forward (5'-3')	Reverse (5'-3')
LASP1	WT 3'UTR	GGAATTCCTGGGGCAGAGAAGTT	CGGATATCCACCCACAGTCTTTTCC
	MT 3'UTR	GGAATTCTGACAACCTGAACA GCACAGCCTCGGGGAT	CGGATATCCACCCACAGTCTTTTCC
pmiR-145p-F1		TTGAGCTCTGGAAGTGGTAAGTGCTG	CAGATCTACTTCCCAAGCTTTTGGGGAG
pmiR-145p-F2		TTGAGCTCCAAAAGCTTGGGAAGTGG	AAGATCTTGTCCCTAAGGACCAGCTG
pmiR-145p-F2A		TTGAGCTCCAAAAGCTTGGGAAGTGG	GAAGATCTTCGGGGTACCCC TGAGATGTAAAGA
pmiR-145p-F2B		CGAGCTCGGGGGTACCCC TCACTTTTAGCAAAGA	GAAGATCTTCGCTCTAGAGCG CCAGAGGGGTGGATT
pmiR-145p-F2C		CGAGCTCGGCTCTAGAGCATGG TTGGTTTAAAT	AAGATCTTGTCCCTAAGGACCAGCTG

Supplementary Table S5: Primer sequences for miR-145 regulatory region analyzed in ChIP assays

Sets of primers	Forward (5'-3')	Reverse (5'-3')
1	AACAATGAGCCCTGGAAAGC	GGACTGGTTTCGGCAATACC
2	AATGAGCAGCAGAGGAGAGC	GGTCCCAGGTTCCATAATCA
3	TTAAAGAAGGCCAAGGCTCA	GGAACTTCAATTGCCCTGAA
4	TGCAGAAGAGAACTCCAGCT	CCAGCTGTGAAACCATGACC
5	CTTCCCACCCCACAGTTG	AAACGCATGCCTGATGGT
